# Tobacco use among adults with disabilities in nine countries–Demographic and Health Survey, 2016–2021

**DOI:** 10.1371/journal.pgph.0003232

**Published:** 2024-06-17

**Authors:** Alissa C. Kress, Aastha Vashist, Qing C. Zhang, Adriana Dragicevic, Gibril J. Njie

**Affiliations:** 1 Office on Smoking and Health, National Center for Chronic Disease Prevention and Health Promotion, Centers for Disease Control and Prevention (CDC), Atlanta, Georgia, United States of America; 2 Noninfectious Disease Programs, CDC Foundation, assigned to the Office on Smoking and Health, National Center for Chronic Disease Prevention and Health Promotion, Centers for Disease Control and Prevention (CDC), Atlanta, Georgia, United States of America; 3 Division of Human Development and Disability, National Center on Birth Defects and Developmental Disabilities, CDC, Atlanta, Georgia, United States of America; Augusta University, UNITED STATES

## Abstract

Few studies have investigated tobacco use among people with disabilities living in low- and middle-income countries (LMICs). We aimed to examine current tobacco use among men and women with disabilities using Demographic and Health Survey (DHS) data from 9 LMICs. We considered a respondent currently use tobacco products if they reported current use of any combustible/smoked tobacco products or smokeless tobacco products. We performed secondary analyses of DHS data from 2016–2021 collected in Haiti, Mali, Mauritania, Nigeria, Pakistan, Rwanda, South Africa, Timor-Leste, and Uganda. We examined marginal effects in logistic regression to calculate the adjusted prevalence and adjusted prevalence differences of tobacco use by disability status, controlling for selected sociodemographic characteristics. The adjusted prevalence of current use of tobacco products among women with a moderate/severe disability, mild disability, and no disability varied across countries, with medians of 1.9% (range = 0.1% [Mali] to 11.3% [Pakistan]), 3.2% (range = 0.9% [Nigeria] to 13.3% [South Africa]), and 2.3% (range = 0.5% [Nigeria] to 8.9% [South Africa]), respectively. For men with moderate/severe disability, the median adjusted prevalence for current use of tobacco products was 18.8% (range = 8.9% [Rwanda] to 55.0% [Timor-Leste]). The median prevalences of current use of tobacco products for men with mild disability and no disability were similar to those with moderate/severe disability, at 16.5% and 15.9%, respectively. Current tobacco product use among people with disabilities varied for countries included in our study; however, with few exceptions, current tobacco product use was similar across disability status groups. Additional research is warranted to determine whether our findings extend beyond the nine countries assessed here. It is important to consider the needs of people with disabilities in tobacco prevention, control, and cessation efforts so that this substantial population can benefit equitably from such programs, interventions, or policies.

## Introduction

In 2021, the World Health Organization (WHO) estimated that approximately 16% of the global population had a disability of some kind, and this number was expected to grow [1]. Disability affects nearly everyone, in some way, at some point in their lives [1]. Disability results from a combination of impairment in body structure or function (e.g., memory loss), activity limitation (e.g., difficulty concentrating), and restriction of participation in normal daily activities (e.g., working) [1, 2]. Health disparities are known to exist among people with disabilities, possibly due to the interplay of factors such as underlying or secondary health conditions, reduced access to health care, and environmental or structural barriers [1, 3, 4]. Disparities experienced by people with disabilities relative to those without disabilities include a higher prevalence of chronic health conditions, such as cardiovascular disease and depression, and health risk behaviors, such as physical inactivity and smoking [1, 5, 6].

Tobacco smoking, smokeless tobacco use, and exposure to secondhand smoke have been linked with disability, chronic diseases, cancers, and death [7, 8]. WHO estimated that 18% of the global population aged 15 years or older smoked tobacco as of 2020 and 6% used smokeless tobacco as of 2018 [9]. In 2019, nearly 7.7 million deaths and 200 million disability-adjusted life years (DALYs) were attributable to tobacco use [10]. Globally, tobacco use varies by country, gender, and socioeconomic status. Among men, prevalence of any tobacco use ranges from 6% in Ghana to 71% in Indonesia; among women, prevalence ranges from 0% in the Democratic People’s Republic of Korea to 53% in Nauru [9]. Globally, there is an association between lower income levels and higher current smoking prevalence, which is consistent across most geographic regions and gender [11].

Several studies using data from high-income countries have examined tobacco use among people with disabilities. In general, findings from these studies demonstrate that tobacco use is higher for people with disabilities than people without disabilities [6, 12–15], although there are some exceptions [16, 17]. Worldwide, more than 80% of people who use tobacco and nearly 80% of people with disabilities live in low- and middle-income countries (LMICs) [1, 18], yet few studies have examined tobacco use among people with disabilities living in LMICs. One study conducted among adults aged 50 years or older in Indonesia found that functional disability was associated with both current and former tobacco use [19]. Another study, conducted in Nigeria, reported a 5.6% prevalence of cigarette smoking among 20- to 40-year-old participants with a disability [20]. Lastly, a study from Rwanda showed that nearly one-third of participants aged 12 to 60 years with lower limb amputation currently smoked or used any tobacco [21].

Worth noting are the inconsistent definitions of both disability and tobacco use across the studies highlighted in the previous paragraph, making it difficult to compare findings across countries and over time. Furthermore, given the paucity of evidence that uses nationally representative data to assess the relationship between disability status and tobacco use using standard disability and tobacco use measures, we aim to examine tobacco use among men and women with disabilities using data from nine LMICs (Haiti, Mali, Mauritania, Nigeria, Pakistan, Rwanda, South Africa, Timor-Leste, and Uganda) that implemented the disability module and assessed tobacco use as part of the Demographic and Health Survey (DHS) between 2016 and 2021.

## Materials and methods

### Data source

We performed secondary analyses of data from 2016–2021 DHS conducted in nine LMICs as defined by the World Bank (Haiti, Mali, Mauritania, Nigeria, Pakistan, Rwanda, South Africa, Timor-Leste, and Uganda) [22]. DHS are nationally representative surveys that collect data on a variety of demographic, health, fertility, and nutrition topics. The surveys are typically conducted among women aged 15–49 years and men aged 15–59 years following stratified two-stage sample designs. Sample weights are developed and provided to enable the generalizability of nationally representative estimates. DHS are implemented by participating countries, with technical assistance provided by USAID through the DHS program [23]. Years of survey implementation vary by country.

DHS is composed of four components: household questionnaire, women’s questionnaire, men’s questionnaire, and biomarker questionnaire. The household questionnaire collects demographic information on members and visitors of each household as well as household characteristics. Beginning with DHS-7 in 2016, countries could also opt to implement a disability module as part of the household questionnaire. The women’s and men’s questionnaires collect information on demographic characteristics, knowledge, and behavior related to health issues such as tobacco use, reproductive health, and other topics. The biomarker questionnaire records height, weight, hemoglobin, and HIV test results; we did not use biomarker data in our analyses.

We selected these nine LMICs for analysis because they implemented the disability module as part of the household questionnaire and assessed tobacco use through the women’s and men’s questionnaires. In Nigeria, the disability module was administered to the 2/3 of households not selected for the men’s questionnaire. Therefore, approximately 2/3 of women were included in the disability module; the men included in the disability module were not surveyed as part of the men’s questionnaire and were excluded from our analyses. In Pakistan, the sampling procedure produced nationally representative weights and separate weights for two provinces—Azad Jammu and Kashmir and Gilgit Baltistan. Because of this, the two provinces were not included in the national estimates and were excluded from our analyses. In South Africa, tobacco use questions were asked as part of an adult health module, which was administered only to women and men in the subsample of households selected for the men’s questionnaire. Therefore, approximately 1/2 of women were surveyed about tobacco use. The number of households interviewed ranged from 9,510 in Mali to 40,427 in Nigeria; household response rates ranged from 83.4% in South Africa to 100% in Rwanda. Table 1 presents additional information on the characteristics of DHS surveys included in our analyses, including sample sizes and ages of respondents surveyed. Data analyses were completed in July 2023.

**Table 1:** Overview of Demographic and Health Survey included in this study, 2016–2021.

	Year of survey	Number of households interviewed	Household response rate	Number of women interviewed	Eligibility criteria for women interviewed	Eligible women response rate	Number of men interviewed	Eligibility criteria for men interviewed	Eligible men response rate
Haiti^a^	2016–2017^b^	13,405	99.7%	14,371	15–49 years	98.9%	9,795	15–64 years	98.0%
Mali	2018	9,510	99.6%	10,519	15–49 years	97.6%	4,618	15–59 years; in selected households^c^	96.1%
Mauritania	2019–2021^b^	11,658	98.7%	15,714	15–49 years	96.2%	5,673	15–59 years; in selected households^c^	91.7%
Nigeria^d^	2018	40,427	99.4%	41,821	15–49 years	99.3%	13,311	15–59 years; in selected households^e^	99.2%
Pakistan^f^	2017–2018^b^	14,540	96.2%	15,068	15–49 years; ever married	94.3%	3,691	15–49 years; ever married; in selected households^e^	86.5%
Rwanda	2019–2020^b^	12,949	100%	14,634	15–49 years	99.7%	6,513	15–59 years; in selected households^c^	99.5%
South Africa^g^	2016	11,083	83.4%	8,514	15–49 years	86.2%	3,618	15–59 years; in selected households^c^	73.1%
Timor-Leste	2016	11,502	98.6%	12,607	15–49 years	97.0%	4,622	15–59 years; in selected households^e^	94.8%
Uganda	2016	19,588	98.2%	18,506	15–49 years	97.0%	5,336	15–54; in selected households^e^	94.0%

^a^ For Haiti, women aged 50–64 years were also interviewed in approximately one-third of households; however, we limited our analyses to women aged 15–49 years for consistency with other countries’ data. Therefore, characteristics of women’s data for Haiti may vary from those shown here.

^b^ Survey implementation occurred across multiple years.

^c^ Approximately one-half of households were selected.

^d^ For Nigeria, the disability module was administered to the two-thirds of households not selected for the men’s questionnaire. Therefore, approximately two-thirds of women were surveyed for the disability module. Men surveyed for the disability module were not surveyed as part of the men’s questionnaire and we therefore were not able to produce tobacco use estimates by disability status for men in Nigeria.

^e^ Approximately one-third of households were selected.

^f^ For Pakistan, the sampling procedure produced weights that are representative at the national level and separately for two provinces (Azad Jammu and Kashmir and Gilgit Baltistan). Therefore, the two provinces were not included in national estimates and were excluded from our analyses.

^g^ For South Africa, tobacco use questions were asked as part of an adult health module, which was administered only to women and men in the subsample of households selected for the men’s questionnaire. Therefore, approximately one-half of women were surveyed about tobacco use.

### Measures

We assessed disability using the Washington Group Short Set on Functioning (WG-SS) question set, which asks whether there is no difficulty, some difficulty, a lot of difficulty, or cannot do at all across six disability domains: vision, hearing, mobility, cognition, self-care, and communication [24]. Using responses to these six questions, variables measuring different thresholds of disability can be created. We chose to create a three-level disability variable (moderate/severe, mild, and no disability). This approach has been used previously [25, 26] and allows for alignment with the WG-SS recommended threshold for measuring disability internationally (i.e., the moderate/severe disability category) [27] while also capturing those with milder disability who may also have accessibility considerations. We considered a respondent to have a moderate/severe disability if they answered a lot of difficulty or cannot do at all for at least one of the disability domains. Respondents who reported some difficulty for at least one disability domain but had no domains for which they reported a lot of difficulty or cannot do at all were considered to have a mild disability. Those who reported no difficulty for all of the disability domains were considered to have no disability. While investigation of disability type is also possible using these questions, it was beyond the scope of the current study.

We assessed tobacco use using selected questions based on Tobacco Questions for Surveys included in DHS, allowing for comparability of tobacco use estimates across countries and time [23, 28]. A respondent was considered to currently use combustible/smoked tobacco products if they reported currently using combustible tobacco products (e.g., cigarettes, kreteks, pipes, cigars, hookah) every day or some days. A respondent was considered to currently use smokeless tobacco products if they reported currently using smokeless tobacco products (e.g., chewing tobacco, snuff, betel quid with tobacco) every day or some days. A respondent was considered to currently use tobacco products if they reported current use of any combustible/smoked tobacco products, smokeless tobacco products, or any other type of tobacco product.

Sociodemographic characteristics included age group (15–24, 25–34, 35–49, and 50–59 years of age), residence (urban, rural), highest level of education attended or completed (no formal education, primary, secondary or higher), employment status (currently employed, not currently employed), wealth index (lowest, lower, middle, higher, highest quintile), marital status (single/never married, married/living in a union, divorced/separated/widowed) and health insurance coverage (yes, no).

### Statistical analyses

We used descriptive statistics to estimate the prevalence and corresponding 95% confidence intervals (CI) for disability status, selected sociodemographic variables (i.e., age, urban/rural residence, level of education, employment status, wealth index, marital status, health insurance coverage), and tobacco use (i.e., current smoked tobacco use, current smokeless tobacco use, current tobacco use). We assessed crude prevalence by tobacco use and disability status. We then examined marginal effects in logistic regression to represent the adjusted prevalence of tobacco use for adults with moderate/severe disability, mild disability, and no disability, along with adjusted prevalence differences (aPD) and the 95% CIs of tobacco use by disability status, controlling for selected sociodemographic characteristics. Positive values for aPD indicate a higher adjusted prevalence for moderate/severe or mild disability than for no disability; negative aPD indicates a higher adjusted prevalence for no disability than for moderate/severe or mild disability. We considered the aPD statistically significant if the p-value was <0.05. We conducted analyses using SAS-callable SUDAAN v11.0.1 (RTI International, Research Triangle Park, North Carolina) accounting for sample weights and other design variables to generate nationally representative estimates. We conducted all analyses separately for men and women and for each country. Responses that were missing or marked as “don’t know” were excluded from the analyses.

### Ethical considerations

DHS survey protocols for data used in this study were approved by the ICF Institutional Review Board. Verbal informed consent was obtained from adult participants and from a parent or guardian of minor participants [29]. This study was not subject to ethical review as it was a secondary analysis of deidentified publicly available data.

## Results

In all nine countries, most women did not report a disability (median = 84.9%) (Table 2). The proportion of women with a moderate/severe disability varied (median = 2.0%), ranging from 0.5% (Nigeria, 95% CI: 0.4%, 0.6%; Timor-Leste, 95% CI: 0.4%, 0.7%) to 4.4% (Pakistan, 95% CI: 3.8%, 5.1%); the proportion with a mild disability (median = 12.7%) ranged from 5.1% (Nigeria, 95% CI: 4.6%, 5.5%) to 16.1% (Pakistan, 95% CI: 14.9%, 17.4%). Nigeria had the lowest proportion of women who reported current use of tobacco products (0.5%, 95% CI: 0.4%, 0.6%), and South Africa had the highest (9.5%, 95% CI: 8.3%, 10.9%). Similar patterns were noted for men, with the majority of men not reporting a disability (median = 83.9%) (Table 3). The proportion of men with a moderate/severe disability ranged from 0.8% (Timor-Leste, 0.5%, 1.2%) to 3.8% (Rwanda, 95% CI: 3.3%, 4.3%) and with a mild disability ranged from 11.1% (South Africa, 95% CI: 9.9%, 12.5%) to 14.8% (Mauritania, 95% CI: 13.4%, 16.3%). Timor-Leste had the highest proportion of men who reported current use of tobacco products (57.6%, 95% CI: 55.3%, 59.9%), with the lowest proportion reported for Nigeria at 6.8% (95% CI: 6.2%, 7.4%).

**Table 2:** Distribution of disability status, selected sociodemographic and other characteristics, and tobacco use, among women aged 15–49 years–Demographic and Health Survey, 2016–2021.

	Haiti % (95% CI)	Mali % (95% CI)	Mauritania % (95% CI)	Nigeria % (95% CI)	Pakistan % (95% CI)	Rwanda % (95% CI)	South Africa % (95% CI)	Timor-Leste % (95% CI)	Uganda % (95% CI)
Disability status									
Moderate/severe disability	1.4 (1.2, 1.7)	1.0 (0.8, 1.3)	2.0 (1.7, 2.3)	0.5 (0.4, 0.6)	4.4 (3.8, 5.1)	3.6 (3.3, 3.9)	2.6 (2.2, 3.0)	0.5 (0.4, 0.7)	2.5 (2.2, 2.8)
Mild disability	14.3 (13.4, 15.2)	10.5 (9.5, 11.6)	14.1 (13.2, 15.1)	5.1 (4.6, 5.5)	16.1 (14.9, 17.4)	14.0 (13.4, 14.7)	11.6 (10.7, 12.6)	7.8 (7.1, 8.5)	12.7 (11.9, 13.5)
No disability	84.3 (83.3, 85.2)	88.5 (87.3, 89.5)	83.9 (82.8, 84.9)	94.4 (94.0, 94.8)	79.5 (78.0, 80.9)	82.4 (81.7, 83.2)	85.6 (84.5, 86.6)	91.7 (91.0, 92.4)	84.9 (84.0, 85.8)
Age group									
15–24 years	41.8 (40.8, 42.9)	38.0 (36.7, 39.3)	40.7 (39.9, 41.6)	36.5 (36.0, 37.1)	20.1 (19.0, 21.3)	38.8 (37.9, 39.7)	33.4 (32.1, 34.7)	40.8 (39.8, 41.9)	43.7 (42.9, 44.5)
25–34 years	29.7 (28.8, 30.7)	34.4 (33.2, 35.5)	30.6 (29.7, 31.6)	32.1 (31.5, 32.7)	40.1 (39.0, 41.2)	28.6 (27.7, 29.5)	32.6 (31.3, 33.9)	30.0 (28.9, 31.1)	30.2 (29.4, 31.1)
35–49 years	28.4 (27.5, 29.3)	27.6 (26.5, 28.7)	28.6 (27.8, 29.4)	31.3 (30.8, 31.9)	39.8 (38.5, 41.0)	32.6 (31.9, 33.3)	34.0 (32.8, 35.3)	29.1 (28.1, 30.2)	26.1 (25.3, 26.8)
Residence									
Urban	46.8 (44.0, 49.7)	26.3 (24.0, 28.7)	51.2 (49.0, 53.5)	45.8 (44.0, 47.6)	36.8 (34.1, 39.6)	19.9 (18.9, 20.9)	67.3 (65.0, 69.5)	33.2 (30.8, 35.7)	26.7 (25.1, 28.4)
Rural	53.2 (50.3, 56.0)	73.7 (71.3, 76.0)	48.8 (46.5, 51.0)	54.2 (52.4, 56.0)	63.2 (60.4, 65.9)	80.1 (79.1, 81.1)	32.7 (30.5, 35.0)	66.8 (64.3, 69.2)	73.3 (71.6, 74.9)
Highest education level attended or completed									
No education	13.3 (12.1, 14.6)	65.5 (63.2, 67.8)	32.8 (30.8, 34.8)	34.9 (33.3, 36.5)	49.2 (46.1, 52.2)	9.4 (8.7, 10.1)	2.0 (1.6, 2.4)	21.7 (20.4, 23.1)	9.6 (8.9, 10.4)
Primary	30.2 (28.8, 31.7)	13.2 (12.2, 14.2)	38.5 (36.7, 40.4)	14.4 (13.8, 15.1)	16.5 (15.3, 17.7)	58.3 (57.1, 59.5)	9.1 (8.2, 10.1)	15.2 (14.3, 16.2)	57.4 (55.9, 58.9)
Secondary or higher	56.5 (54.2, 58.6)	21.3 (19.3, 23.4)	28.7 (26.8, 30.6)	50.6 (49.1, 52.2)	34.3 (31.4, 37.4)	32.3 (31.0, 33.7)	88.9 (87.7, 90.0)	63.0 (61.3, 64.7)	32.9 (31.3, 34.6)
Employment status									
Currently employed	43.7 (42.5, 45.0)	54.6 (51.9, 57.3)	20.1 (18.8, 21.5)	65.0 (64.1, 65.9)	17.3 (15.7, 18.9)	66.3 (65.2, 67.4)	34.4 (32.6, 36.3)	33.7 (31.9, 35.6)	73.1 (71.9, 74.2)
Not currently employed	56.3 (55.0, 57.5)	45.4 (42.7, 48.1)	79.9 (78.5, 81.2)	35.0 (34.1, 35.9)	82.7 (81.1, 84.3)	33.7 (32.6, 34.8)	65.6 (63.7, 67.4)	66.3 (64.4, 68.1)	26.9 (25.8, 28.1)
Wealth index									
Lowest	15.1 (13.3, 17.1)	17.5 (15.4, 19.9)	17.2 (15.2, 19.5)	17.3 (16.0, 18.6)	18.3 (15.7, 21.1)	18.7 (17.5, 20.0)	19.4 (16.8, 22.2)	16.5 (14.8, 18.4)	17.5 (16.2, 19.0)
Lower	16.9 (15.3, 18.6)	18.8 (17.1, 20.7)	18.2 (16.5, 20.0)	19.2 (18.0, 20.6)	19.7 (17.6, 21.8)	18.8 (17.8, 19.9)	20.1 (18.2, 22.2)	18.1 (16.8, 19.5)	18.4 (17.3, 19.5)
Middle	19.3 (17.3, 21.4)	19.1 (17.5, 20.8)	20.0 (18.2, 22.0)	19.6 (18.5, 20.8)	20.3 (18.5, 22.1)	18.8 (17.9, 19.8)	21.2 (19.2, 23.3)	19.2 (17.8, 20.7)	18.7 (17.6, 19.8)
Higher	23.6 (21.5, 25.9)	21.1 (18.6, 23.7)	22.1 (20.1, 24.3)	21.5 (20.0, 23.1)	21.0 (18.8, 23.3)	20.3 (19.1, 21.5)	20.7 (18.6, 23.0)	22.0 (20.4, 23.7)	19.9 (18.7, 21.2)
Highest	25.1 (22.2, 28.2)	23.4 (21.0, 26.0)	22.5 (19.9, 25.3)	22.4 (20.8, 24.0)	20.9 (18.5, 23.4)	23.3 (21.6, 25.1)	18.6 (16.0, 21.5)	24.1 (22.3, 26.1)	25.5 (23.3, 27.8)
Marital status									
Single/never married	40.5 (39.2, 41.9)	16.0 (14.6, 17.3)	26.6 (25.6, 27.7)	25.2 (24.3, 26.1)	0.0 (N/A)	40.4 (39.4, 41.5)	58.6 (57.0, 60.2)	36.6 (35.4, 37.9)	25.8 (24.9, 26.8)
Married/living in a union	51.5 (50.0, 53.0)	81.4 (80.0, 82.8)	62.5 (61.2, 63.8)	69.6 (68.6, 70.5)	95.7 (95.1, 96.2)	50.6 (49.5, 51.6)	35.8 (34.2, 37.4)	61.1 (59.8, 62.3)	60.6 (59.6, 61.7)
Divorced/separated/widowed	8.0 (7.3, 8.7)	2.6 (2.2, 3.1)	10.8 (10.2, 11.6)	5.2 (4.9, 5.6)	4.3 (3.8, 4.9)	9.0 (8.5, 9.6)	5.5 (5.0, 6.2)	2.3 (2.1, 2.6)	13.5 (12.9, 14.2)
Health insurance coverage									
Yes	2.9 (2.5, 3.4)	5.3 (4.1, 6.8)	8.7 (7.7, 9.9)	2.6 (2.3, 3.1)	1.4 (1.1, 1.8)	82.6 (81.5, 83.7)	--^a^	--^a^	1.4 (1.1, 1.7)
No	97.1 (96.6, 97.5)	94.7 (93.2, 95.9)	91.3 (90.1, 92.3)	97.4 (96.9, 97.7)	98.6 (98.2, 98.9)	17.4 (16.3, 18.5)	--^a^	--^a^	98.6 (98.3, 98.9)
Currently use combustible/smoked tobacco products									
Yes	1.7 (1.4, 2.1)	0.7 (0.5, 0.9)	2.0 (1.7, 2.3)	0.3 (0.2, 0.4)	4.7 (4.0, 5.5)	0.9 (0.7, 1.1)	6.8 (5.7, 8.1)	4.1 (3.5, 4.8)	0.8 (0.7, 1.0)
No	98.3 (97.9, 98.6)	99.3 (99.1, 99.5)	98.0 (97.7, 98.3)	99.7 (99.6, 99.8)	95.3 (94.5, 96.0)	99.1 (98.9, 99.3)	93.2 (91.9, 94.3)	95.9 (95.2, 96.5)	99.2 (99.0, 99.3)
Currently use smokeless tobacco products									
Yes	2.9 (2.4, 3.5)	0.2 (0.1, 0.3)	0.5 (0.4, 0.7)	0.1 (0.0, 0.2)	3.4 (2.7, 4.3)	0.8 (0.7, 1.0)	2.3 (1.7, 2.9)	0.2 (0.1, 0.3)	0.6 (0.5, 0.8)
No	97.1 (96.5, 97.6)	99.8 (99.7, 99.9)	99.5 (99.3, 99.6)	99.9 (99.8, 100)	96.6 (95.7, 97.3)	99.2 (99.0, 99.3)	97.7 (97.1, 98.3)	99.8 (99.7, 99.9)	99.4 (99.2, 99.5)
Currently use tobacco products^b^									
Yes	4.5 (3.9, 5.2)	1.0 (0.8, 1.3)	2.4 (2.1, 2.8)	0.5 (0.4, 0.6)	7.8 (6.8, 9.0)	1.5 (1.3, 1.7)	9.5 (8.3, 10.9)	4.7 (4.1, 5.3)	1.6 (1.4, 1.9)
No	95.5 (94.8, 96.1)	99.0 (98.7, 99.2)	97.6 (97.2, 97.9)	99.5 (99.4, 99.6)	92.2 (91.0, 93.2)	98.5 (98.3, 98.7)	90.5 (89.1, 91.7)	95.3 (94.7, 95.9)	98.4 (98.1, 98.6)

Abbreviation: CI–confidence interval

^a^ Data not available.

^b^ Current use of tobacco products includes combustible/smoked tobacco products, smokeless tobacco products, and any other type of tobacco use included in the Demographic and Health Survey.

**Table 3:** Distribution of disability status, selected sociodemographic and other characteristics, and tobacco use, among men aged 15–59 years–Demographic and Health Survey, 2016–2021.

	Haiti % (95% CI)	Mali % (95% CI)	Mauritania % (95% CI)	Nigeria % (95% CI)	Pakistan % (95% CI)	Rwanda % (95% CI)	South Africa % (95% CI)	Timor-Leste % (95% CI)	Uganda % (95% CI)
Disability status									
Moderate/severe disability	2.1 (1.7, 2.4)	1.9 (1.5, 2.6)	2.0 (1.5, 2.6)	--^a^	3.3 (2.5, 4.4)	3.8 (3.3, 4.3)	2.9 (2.3, 3.6)	0.8 (0.5, 1.2)	2.6 (2.1, 3.2)
Mild disability	13.9 (13.1, 14.8)	13.0 (11.7, 14.5)	14.8 (13.4, 16.3)	--^a^	12.9 (11.1, 14.9)	13.4 (12.5, 14.3)	11.1 (9.9, 12.5)	13.5 (12.4, 14.7)	14.0 (12.8, 15.2)
No disability	84.0 (83.1, 84.9)	85.0 (83.4, 86.6)	83.2 (81.7, 84.6)	--^a^	83.8 (81.4, 85.9)	82.9 (81.9, 83.9)	86.0 (84.5, 87.4)	85.7 (84.4, 86.9)	83.4 (82.1, 84.7)
Age group									
15–24 years	38.7 (37.4, 40.1)	31.2 (29.3, 33.1)	35.4 (33.4, 37.3)	29.2 (28.1, 30.3)	9.7 (8.4, 11.2)	38.2 (36.9, 39.4)	34.1 (31.8, 36.5)	36.6 (34.7, 38.5)	41.9 (40.2, 43.7)
25–34 years	24.9 (23.8, 26.0)	24.6 (23.0, 26.3)	23.3 (21.7, 25.0)	25.5 (24.5, 26.5)	38.5 (36.0, 40.9)	23.7 (22.5, 25.0)	26.4 (24.5, 28.4)	23.7 (21.5, 26.0)	27.7 (26.2, 29.2)
35–49 years	23.7 (22.6, 24.7)	31.5 (29.8, 33.3)	28.4 (26.7, 30.2)	34.5 (33.4, 35.5)	51.8 (49.2, 54.4)	27.9 (26.7, 29.0)	27.9 (25.8, 30.1)	27.9 (26.3, 29.6)	24.8 (23.5, 26.1)
50–59 years	12.8 (11.9, 13.6)	12.7 (11.8, 13.8)	13.0 (11.9, 14.1)	10.8 (10.2, 11.5)	--^a^	10.2 (9.5, 11.0)	11.5 (10.3, 12.9)	11.8 (10.7, 13.1)	5.6 (5.0, 6.3)
Residence									
Urban	42.4 (39.7, 45.2)	25.6 (23.1, 28.1)	62.4 (59.6, 65.2)	46.4 (44.7, 48.2)	40.2 (36.8, 43.6)	18.8 (17.5, 20.2)	68.9 (66.4, 71.3)	31.9 (29.3, 34.6)	24.9 (23.0, 27.0)
Rural	57.6 (54.8, 60.3)	74.4 (71.9, 76.9)	37.6 (34.8, 40.4)	53.6 (51.8, 55.3)	59.8 (56.4, 63.2)	81.2 (79.8, 82.5)	31.1 (28.7, 33.6)	68.1 (65.4, 70.7)	75.1 (73.0, 77.0)
Highest education level attended or completed									
No education	14.8 (13.6, 16.0)	55.4 (52.8, 58.0)	33.2 (31.0, 35.5)	22.6 (21.1, 24.1)	25.4 (22.7, 28.4)	9.0 (8.2, 9.8)	3.0 (2.4, 3.7)	22.7 (20.8, 24.8)	4.2 (3.4, 5.1)
Primary	29.4 (27.9, 30.9)	14.4 (13.1, 15.8)	32.0 (29.8, 34.2)	14.4 (13.5, 15.3)	20.3 (18.4, 22.4)	61.5 (59.9, 63.1)	14.2 (12.8, 15.9)	19.0 (17.5, 20.5)	55.3 (53.3, 57.2)
Secondary or higher	55.9 (53.6, 58.1)	30.2 (27.8, 32.7)	34.8 (32.5, 37.3)	63.1 (61.4, 64.7)	54.2 (51.1, 57.4)	29.6 (28.0, 31.2)	82.8 (80.9, 84.5)	58.3 (56.0, 60.5)	40.5 (38.5, 42.6)
Employment status									
Currently employed	69.8 (68.2, 71.3)	89.9 (88.1, 91.4)	61.3 (58.7, 63.9)	87.1 (86.3, 87.9)	96.2 (95.1, 97.0)	87.7 (86.5, 88.7)	46.7 (44.3, 49.1)	71.7 (69.6, 73.7)	92.3 (91.3, 93.1)
Not currently employed	30.2 (28.7, 31.8)	10.1 (8.6, 11.9)	38.7 (36.1, 41.3)	12.9 (12.1, 13.7)	3.8 (3.0, 4.9)	12.3 (11.3, 13.5)	53.3 (50.9, 55.7)	28.3 (26.3, 30.4)	7.7 (6.9, 8.7)
Wealth index									
Lowest	17.4 (15.4, 19.6)	18.6 (16.1, 21.4)	12.8 (10.9, 15.1)	17.1 (15.7, 18.6)	17.6 (14.8, 20.9)	15.6 (14.4, 17.0)	19.4 (16.3, 22.8)	16.4 (14.3, 18.8)	16.9 (15.2, 18.6)
Lower	17.9 (16.3, 19.6)	19.9 (17.7, 22.3)	14.5 (12.7, 16.4)	17.9 (16.6, 19.4)	19.5 (16.9, 22.3)	18.8 (17.3, 20.3)	20.6 (18.4, 23.1)	20.7 (18.9, 22.6)	17.7 (16.4, 19.2)
Middle	20.8 (18.7, 23.0)	18.6 (16.7, 20.6)	19.5 (17.2, 22.2)	20.0 (18.6, 21.4)	19.7 (17.2, 22.4)	21.1 (19.7, 22.7)	21.5 (19.0, 24.3)	20.3 (18.1, 22.5)	19.6 (18.1, 21.3)
Higher	20.8 (18.9, 22.8)	20.1 (17.5, 23.0)	24.8 (22.3, 27.5)	21.7 (19.9, 23.6)	21.6 (19.0, 24.5)	21.9 (20.3, 23.5)	20.6 (17.9, 23.6)	20.6 (18.8, 22.6)	22.0 (20.3, 23.9)
Highest	23.2 (20.4, 26.3)	22.8 (20.1, 25.6)	28.3 (25.0, 31.8)	23.3 (21.6, 25.0)	21.6 (18.5, 25.1)	22.6 (20.7, 24.5)	17.9 (15.1, 21.1)	22.0 (19.8, 24.4)	23.7 (21.3, 26.3)
Marital status									
Single/never married	47.4 (46.0, 48.9)	33.1 (31.2, 35.1)	46.0 (44.1, 47.9)	37.3 (36.1, 38.5)	0.0 (N/A)	44.1 (42.9, 45.6)	59.2 (56.7, 61.7)	45.0 (43.1, 46.9)	39.0 (37.3, 40.8)
Married/living in a union	46.4 (45.0, 47.7)	66.1 (64.0, 68.0)	51.7 (49.8, 53.7)	61.5 (60.2, 62.7)	98.1 (97.3, 98.6)	53.4 (52.0, 54.9)	35.3 (33.0, 37.6)	54.0 (52.1, 55.9)	55.4 (53.5, 57.2)
Divorced/separated/widowed	6.2 (5.6, 6.9)	0.8 (0.6, 1.2)	2.3 (1.8, 2.9)	1.3 (1.1, 1.5)	1.9 (1.4, 2.7)	2.3 (1.9, 2.8)	5.5 (4.5, 6.7)	1.0 (0.7, 1.4)	5.6 (4.9, 6.4)
Health insurance coverage									
Yes	4.8 (4.0, 5.8)	6.2 (5.1, 7.5)	8.7 (7.3, 10.3)	3.4 (2.9, 3.9)	4.1 (3.1, 5.4)	83.3 (81.9, 84.6)	--^a^	--^a^	1.9 (1.4, 2.6)
No	95.2 (94.2, 96.0)	93.8 (92.5, 94.9)	91.3 (89.7, 92.7)	96.6 (96.1, 97.1)	95.9 (94.6, 96.9)	16.7 (15.4, 18.1)	--^a^	--^a^	98.1 (97.4, 98.6)
Currently use combustible/smoked tobacco products									
Yes	9.8 (9.0, 10.7)	12.7 (11.5, 14.0)	16.1 (14.5, 17.8)	5.5 (5.1, 6.1)	22.6 (20.4, 25.0)	8.8 (8.0, 9.6)	38.3 (36.1, 40.6)	53.7 (51.4, 56.0)	10.1 (9.1, 11.2)
No	90.2 (89.3, 91.0)	87.3 (86.0, 88.5)	83.9 (82.2, 85.5)	94.5 (93.9, 94.9)	77.4 (75.0, 79.6)	91.2 (90.4, 92.0)	61.7 (59.4, 63.9)	46.3 (44.0, 48.6)	89.9 (88.8, 90.9)
Currently use smokeless tobacco products									
Yes	3.6 (3.0, 4.3)	1.6 (1.2, 2.2)	1.6 (1.1, 2.1)	1.5 (1.2, 1.8)	14.6 (12.4, 17.0)	0.1 (0.0, 0.2)	0.9 (0.5, 1.5)	21.9 (20.0, 23.9)	1.3 (1.0, 1.7)
No	96.4 (95.7, 97.0)	98.4 (97.8, 98.8)	98.4 (97.9, 98.9)	98.5 (98.2, 98.8)	85.4 (83.0, 87.6)	99.9 (99.8, 100)	99.1 (98.5, 99.5)	78.1 (76.1, 80.0)	98.7 (98.3, 99.0)
Currently use tobacco products^b^									
Yes	12.6 (11.5, 13.7)	14.9 (13.6, 16.3)	17.3 (15.7, 19.0)	6.8 (6.2, 7.4)	34.6 (31.9, 37.5)	8.8 (8.1, 9.7)	39.1 (36.8, 41.4)	57.6 (55.3, 59.9)	11.0 (10.0, 12.2)
No	87.4 (86.3, 88.5)	85.1 (83.7, 86.4)	82.7 (81.0, 84.3)	93.2 (92.6, 93.8)	65.4 (62.5, 68.1)	91.2 (90.3, 91.9)	60.9 (58.6, 63.2)	42.4 (40.1, 44.7)	89.0 (87.8, 90.0)

Abbreviation: CI–confidence interval

^a^ Data not available.

^b^ Current use of tobacco products includes combustible/smoked tobacco products, smokeless tobacco products, and any other type of tobacco use included in the Demographic and Health Survey.

### Current use of smoked tobacco products

Table 4 displays the crude and adjusted prevalence and adjusted prevalence differences for tobacco use by disability status among women. Among women with a moderate/severe disability, the median adjusted prevalence of current use of smoked tobacco products was 1.7%; Pakistan had the highest adjusted prevalence (6.8%, 95% CI: 4.2%, 10.9%) and Nigeria had the lowest (0.0%). For women with mild disability, the adjusted prevalence of current tobacco smoking ranged from 0.4% (95% CI: 0.2%, 0.9%) for Mali to 11.3% (95% CI: 8.0%, 15.9%) for South Africa (median = 2.2%). Moreover, South Africa also had the highest proportion of women who currently smoked tobacco among women with no disability (6.2%, 95% CI: 5.1%, 7.5%) while Nigeria had the lowest at 0.3% (95% CI: 0.2%, 0.5%) (median = 1.6%). Relative to women with no disability, there was no significant difference in the adjusted prevalence of current use of smoked tobacco products among those with a moderate/severe disability in 87.5% of the countries. In Mali, women with no disability had a higher prevalence of current use of smoked tobacco products relative to women with moderate/severe disability (aPD: -0.7 percentage points [pct pts]). For women with mild disability, the prevalence of current tobacco smoking was significantly higher relative to women with no disability in South Africa (aPD: 5.2 pct pts) and Timor-Leste (aPD: 2.8 pct pts). There was no significant difference in the prevalence of current use of smoked tobacco products between the two groups in the remaining seven countries.

**Table 4:** Crude prevalence, adjusted prevalence,^a^ and adjusted prevalence difference^a^ of tobacco use by disability status among women aged 15–49 years–Demographic and Health Survey, 2016–2021.

Tobacco use indicator	Country	Moderate/severe disability		Mild disability		No disability		Adjusted prevalence difference^b^	
		Crude % (95% CI)	Adj % (95% CI)	Crude % (95% CI)	Adj % (95% CI)	Crude % (95% CI)	Adj % (95% CI)	Moderate/severe vs. no disability	Mild disability vs. no disability
Currently use combustible/smoked tobacco products									
	Haiti	4.5 (2.2, 9.1)	3.6 (1.8, 6.9)	2.8 (2.0, 3.8)	2.2 (1.5, 3.1)	1.5 (1.3, 1.8)	1.6 (1.3, 1.9)	2.0 (-0.4, 4.4)	0.6 (-0.2, 1.3)
	Mali	<0.01 (0.0, 0.0)	<0.01 (0.0, 0.0)	0.4 (0.2, 0.9)	0.4 (0.2, 0.9)	0.7 (0.5, 1.0)	0.7 (0.5, 1.0)	-0.7 (-1.0, -0.5)^c^	-0.3 (-0.7, 0.1)
	Mauritania	2.2 (0.7, 7.3)	1.7 (0.5, 5.7)	3.4 (2.5, 4.8)	2.5 (1.8, 3.4)	1.7 (1.5, 2.1)	1.9 (1.6, 2.2)	-0.2 (-2.3, 2.0)	0.6 (-0.2, 1.4)
	Nigeria	0.0 (N/A)^d^	0.0 (N/A)^d^	0.8 (0.4, 1.6)	0.6 (0.2, 1.3)	0.3 (0.2, 0.5)	0.3 (0.2, 0.5)	N/A^d^	0.3 (-0.2, 0.7)
	Pakistan	7.5 (4.7, 11.8)	6.8 (4.2, 10.9)	4.7 (3.4, 6.3)	4.4 (3.2, 6.0)	4.6 (3.8, 5.4)	4.7 (3.9, 5.5)	2.2 (-1.0, 5.4)	-0.3 (-1.7, 1.1)
	Rwanda	1.0 (0.4, 2.3)	0.6 (0.2, 1.4)	1.5 (1.1, 2.2)	1.0 (0.7, 1.5)	0.7 (0.6, 0.9)	0.8 (0.6, 1.1)	-0.2 (-0.8, 0.3)	0.2 (-0.2, 0.6)
	South Africa	5.9 (2.7, 12.3)	6.5 (3.0, 13.5)	11.5 (7.9, 16.5)	11.3 (8.0, 15.9)	6.2 (5.1, 7.6)	6.2 (5.1, 7.5)	0.4 (-4.6, 5.3)	5.2 (1.2, 9.1)^c^
	Timor-Leste	4.6 (1.5, 13.4)	3.7 (1.2, 10.8)	9.5 (7.3, 12.3)	6.6 (5.1, 8.5)	3.6 (3.1, 4.3)	3.8 (3.2, 4.5)	-0.1 (-4.2, 3.9)	2.8 (1.2, 4.4)^c^
	Uganda	1.9 (1.0, 3.6)	1.3 (0.7, 2.5)	1.3 (0.9, 1.8)	0.9 (0.6, 1.3)	0.7 (0.6, 0.9)	0.8 (0.6, 1.0)	0.5 (-0.4, 1.4)	0.1 (-0.3, 0.5)
Currently use smokeless tobacco products									
	Haiti	6.1 (3.0, 12.1)	4.7 (2.3, 9.5)	5.5 (4.1, 7.2)	4.1 (3.1, 5.4)	2.4 (2.0, 2.9)	2.6 (2.1, 3.1)	2.1 (-1.2, 5.4)	1.5 (0.6, 2.5)^c^
	Mali	0.1 (0.0, 0.4)	2.7 (1.1, 6.5)	0.2 (0.0, 0.9)	2.8 (1.2, 6.5)	0.2 (0.1, 0.3)	2.8 (2.3, 3.4)	-0.1 (-2.5, 2.3)	0.0 (-2.7, 2.6)
	Mauritania	1.6 (0.3, 7.8)	1.1 (0.2, 5.8)	0.9 (0.5, 1.5)	0.7 (0.4, 1.1)	0.4 (0.3, 0.6)	0.4 (0.3, 0.6)	0.7 (-1.2, 2.6)	0.2 (-0.1, 0.6)
	Nigeria	0.8 (0.1, 5.6)	26.8 (14.4, 44.3)	0.1 (0.0, 0.5)	26.5 (15.7, 41.3)	0.1 (0.0, 0.2)	26.6 (24.1, 29.2)	0.2 (-16.0, 16.9)	0.0 (-14.0, 14.1)
	Pakistan	5.8 (3.7, 9.0)	4.6 (2.9, 7.1)	3.9 (2.8, 5.5)	3.3 (2.4, 4.6)	3.1 (2.4, 4.1)	3.3 (2.5, 4.3)	1.2 (-0.7, 3.2)	0.0 (-1.2, 1.2)
	Rwanda	2.2 (1.2, 3.9)	17.5 (12.0, 24.8)	1.4 (1.0, 2.0)	17.0 (13.7, 20.8)	0.7 (0.5, 0.8)	16.9 (15.7, 18.2)	0.6 (-6.0, 7.2)	0.0 (-3.8, 3.9)
	South Africa	3.8 (1.8, 8.1)	2.2 (1.0, 4.8)	3.6 (1.8, 6.8)	2.4 (1.3, 4.4)	2.0 (1.6, 2.6)	2.2 (1.7, 2.9)	0.0 (-1.9, 1.8)	0.1 (-1.2, 1.5)
	Timor-Leste	1.5 (0.2, 9.9)	19.4 (6.0, 47.6)	0.7 (0.3, 1.9)	18.7 (11.0, 30.1)	0.2 (0.1, 0.3)	18.5 (17.1, 19.9)	0.9 (-20.0, 21.8)	0.3 (-9.4, 9.9)
	Uganda	0.4 (0.1, 1.2)	0.3 (0.1, 1.0)	0.3 (0.2, 0.6)	0.2 (0.1, 0.4)	0.6 (0.5, 0.8)	0.7 (0.5, 0.9)	-0.4 (-0.8, -0.1)^c^	-0.5 (-0.7, -0.3)^c^
Currently use tobacco products^e^									
	Haiti	10.1 (6.1, 16.4)	8.0 (4.9, 12.7)	7.8 (6.2, 9.7)	6.0 (4.8, 7.5)	3.8 (3.3, 4.5)	4.0 (3.5, 4.7)	3.9 (0.3, 7.6)^c^	2.0 (0.7, 3.2)^c^
	Mali	0.1 (0.0, 0.4)	0.1 (0.0, 0.3)	1.1 (0.5, 2.1)	1.0 (0.5, 1.9)	1.1 (0.8, 1.4)	1.1 (0.8, 1.4)	-1.0 (-1.3, -0.7)^c^	-0.1 (-0.7, 0.5)
	Mauritania	2.4 (0.8, 7.2)	1.9 (0.6, 5.6)	4.4 (3.3, 5.9)	3.1 (2.3, 4.2)	2.1 (1.8, 2.5)	2.3 (2.0, 2.7)	-0.4 (-2.6, 1.8)	0.9 (-0.1, 1.8)
	Nigeria	0.8 (0.1, 5.6)	0.5 (0.1, 3.1)	1.0 (0.5, 1.8)	0.7 (0.4, 1.3)	0.5 (0.4, 0.7)	0.5 (0.4, 0.7)	0.0 (-0.9, 0.9)	0.1 (-0.3, 0.6)
	Pakistan	13.2 (9.2, 18.5)	11.2 (7.7, 16.1)	8.3 (6.5, 10.4)	7.4 (5.8, 9.4)	7.4 (6.4, 8.6)	7.7 (6.6, 8.9)	3.5 (-0.6, 7.7)	-0.3 (-2.1, 1.5)
	Rwanda	2.5 (1.5, 4.3)	1.6 (0.9, 2.7)	2.7 (2.0, 3.5)	1.7 (1.3, 2.2)	1.2 (1.0, 1.5)	1.4 (1.2, 1.7)	0.2 (-0.7, 1.1)	0.3 (-0.3, 0.8)
	South Africa	10.7 (6.4, 17.2)	9.9 (6.0, 16.0)	14.7 (11.0, 19.4)	13.2 (9.9, 17.3)	8.7 (7.4, 10.2)	8.9 (7.6, 10.3)	1.1 (-4.0, 6.1)	4.3 (0.5, 8.1)^c^
	Timor-Leste	5.4 (2.0, 14.0)	4.3 (1.5, 11.3)	11.0 (8.7, 13.8)	7.6 (6.0, 9.5)	4.1 (3.5, 4.8)	4.3 (3.7, 5.0)	-0.0 (-4.3, 4.3)	3.3 (1.6, 5.0)^c^
	Uganda	2.8 (1.7, 4.7)	1.9 (1.1, 3.3)	1.7 (1.2, 2.3)	1.2 (0.9, 1.6)	1.5 (1.3, 1.8)	1.7 (1.4, 2.0)	0.2 (-0.8, 1.3)	-0.5 (-0.9, -0.1)^c^

Abbreviation: CI–confidence interval

^a^ Adjusted for: age group, residence, education level, employment status, wealth index, marital status, and health insurance coverage.

^b^ Positive values for adjusted prevalence differences (aPD) indicate higher adjusted prevalence for moderate/severe or mild disability than for no disability; negative aPD indicates higher adjusted prevalence for no disability than for moderate/severe or mild disability.

^c^ Difference significant at p<0.05.

^d^ Estimate for current use of combustible/smoked tobacco among women with moderate/severe disability in Nigeria was 0.0; therefore, no CIs or aPD were calculated.

^e^ Current use of tobacco products includes combustible/smoked tobacco products, smokeless tobacco products, and any other type of tobacco use included in the Demographic and Health Survey.

Across eight countries with available data for men, the median adjusted prevalence for men with moderate/severe disability who reported current use of smoked tobacco products was 17.8%, ranging from 8.5% for Rwanda (95% CI: 5.3%, 13.2%) to 45.7% for South Africa (95% CI: 37.4%, 54.2%) (Table 5). For men with mild disability, the median adjusted prevalence was 14.3% (range = 8.5% [Rwanda; 95% CI: 7.2%, 10.1%], 47.4% [Timor-Leste; 95% CI: 42.7%, 52.1%]). The median adjusted prevalence of current use of smoked tobacco products for men with no disability was similar to that of men with mild disability at 14.3%, ranging from 8.9% for Rwanda (95% CI: 8.0%, 9.9%) to 54.8% for Timor-Leste (95% CI: 52.4%, 57.2%). Compared with men with no disability, those with moderate/severe disability did not have a higher prevalence of current use of smoked tobacco products in the eight countries assessed. However, in Timor-Leste, men with no disability had a significantly higher prevalence of current use of smoked tobacco products than men with a mild disability (aPD: -7.4 pct pts).

**Table 5:** Crude prevalence, adjusted prevalence,^a^ and adjusted prevalence difference^a^ of tobacco use by disability status among men aged 15–59 years–Demographic and Health Survey, 2016–2021.

Tobacco use indicator	Country	Moderate/severe disability		Mild disability		No disability		Adjusted prevalence difference^b^	
		Crude % (95% CI)	Adj % (95% CI)	Crude % (95% CI)	Adj % (95% CI)	Crude % (95% CI)	Adj % (95% CI)	Moderate/severe vs. no disability	Mild disability vs. no disability
Currently use combustible/smoked tobacco products									
	Haiti	13.7 (9.3, 19.7)	9.7 (6.2, 15.0)	14.2 (12.1, 16.7)	9.5 (7.9, 11.4)	9.0 (8.1, 9.9)	9.2 (8.3, 10.1)	0.6 (-3.9, 5.0)	0.3 (-1.6, 2.3)
	Mali	13.2 (7.0, 23.5)	12.0 (6.5, 21.2)	14.1 (11.2, 17.5)	12.6 (10.0, 15.8)	12.5 (11.2, 13.9)	12.7 (11.4, 14.2)	-0.7 (-7.9, 6.5)	-0.1 (-3.3, 3.1)
	Mauritania	21.4 (9.9, 40.0)	23.5 (11.2, 42.7)	15.9 (12.8, 19.5)	15.9 (12.8, 19.5)	16.0 (14.3, 17.8)	15.9 (14.3, 17.8)	7.6 (-8.1, 23.3)	0.0 (-3.7, 3.6)
	Nigeria	--^c^	--^c^	--^c^	--^c^	--^c^	--^c^	--^c^	--^c^
	Pakistan	34.8 (24.2, 47.0)	29.9 (21.1, 40.5)	24.3 (18.7, 30.8)	20.2 (15.3, 26.2)	21.9 (19.5, 24.5)	22.7 (20.3, 25.4)	7.2 (-2.8, 17.1)	-2.5 (-8.5, 3.5)
	Rwanda	13.4 (9.6, 18.5)	8.6 (6.3, 11.6)	13.4 (11.3, 15.9)	8.5 (7.2, 10.1)	7.8 (7.0, 8.7)	8.9 (8.0, 9.9)	-0.3 (-3.1, 2.5)	-0.3 (-2.0, 1.4)
	South Africa	48.6 (39.9, 57.4)	45.7 (37.4, 54.2)	37.6 (31.8, 43.8)	35.4 (29.6, 41.7)	38.0 (35.5, 40.4)	38.3 (35.9, 40.8)	7.3 (-1.4, 16.0)	-2.9 (-9.5, 3.7)
	Timor-Leste	50.3 (31.8, 68.8)	45.2 (27.4, 64.3)	54.0 (49.0, 59.0)	47.4 (42.7, 52.1)	53.7 (51.2, 56.1)	54.8 (52.4, 57.2)	-9.6 (-29.0, 9.5)	-7.4 (-13.0, -2.3)^d^
	Uganda	13.9 (8.8, 21.3)	8.5 (5.3, 13.2)	15.1 (12.4, 18.1)	11.6 (9.5, 14.2)	9.2 (8.2, 10.4)	9.9 (8.8, 11.2)	-1.5 (-5.5, 2.6)	1.7 (-0.8, 4.2)
Currently use smokeless tobacco products									
	Haiti	6.6 (3.7, 11.4)	4.3 (2.3, 7.9)	6.9 (5.3, 9.0)	3.9 (2.8, 5.4)	3.0 (2.4, 3.6)	3.3 (2.6, 4.0)	1.0 (-1.6, 3.7)	0.6 (-0.6, 1.9)
	Mali	4.3 (1.6, 11.4)	2.6 (0.9, 7.5)	2.9 (1.8, 4.8)	2.1 (1.3, 3.3)	1.4 (1.0, 1.9)	1.5 (1.1, 2.1)	1.1 (-1.8, 4.0)	0.6 (-0.5, 1.7)
	Mauritania	0.0 (N/A)^e^	0.0 (N/A)^e^	1.3 (0.6, 2.7)	1.2 (0.5, 2.5)	1.6 (1.2, 2.3)	1.7 (1.2, 2.4)	N/A^e^	-0.5 (-1.6, 0.6)
	Nigeria	--^c^	--^c^	--^c^	--^c^	--^c^	--^c^	--^c^	--^c^
	Pakistan	14.8 (8.3, 25.1)	14.7 (8.3, 24.8)	17.2 (12.9, 22.7)	17.1 (12.8, 22.5)	14.2 (12.0, 16.7)	14.2 (12.0, 16.7)	0.6 (-7.7, 8.9)	2.9 (-1.8, 7.6)
	Rwanda	0.1 (0.0, 0.5)	19.1 (5.3, 50.1)	0.1 (0.0, 0.9)	19.2 (7.7, 40.4)	0.1 (0.0, 0.2)	19.2 (18.0, 20.5)	-0.1 (-22.0, 22.4)	0.0 (-17.0, 17.2)
	South Africa	0.9 (0.2, 3.6)	0.9 (0.2, 3.9)	0.9 (0.3, 2.2)	0.8 (0.3, 1.9)	0.9 (0.5, 1.6)	0.9 (0.5, 1.6)	0.0 (-1.4, 1.4)	-0.1 (-1.0, 0.7)
	Timor-Leste	32.1 (15.8, 54.3)	27.3 (14.9, 44.5)	26.9 (22.3, 32.1)	23.7 (19.5, 28.4)	21.0 (19.1, 23.0)	21.5 (19.6, 23.6)	5.7 (-9.4, 20.9)	2.2 (-2.4, 6.7)
	Uganda	0.2 (0.0, 1.2)	1.0 (0.3, 3.2)	0.8 (0.3, 1.8)	1.6 (0.9, 2.9)	1.4 (1.1, 1.9)	2.5 (2.0, 3.0)	-1.4 (-2.7, -0.1)^d^	-0.9 (-1.9, 0.2)
Currently use tobacco products^f^									
	Haiti	19.7 (14.1, 26.9)	14.3 (9.8, 20.4)	19.5 (16.9, 22.4)	12.6 (10.7, 14.8)	11.3 (10.2, 12.4)	11.8 (10.7, 13.0)	2.6 (-2.8, 7.9)	0.9 (-1.3, 3.0)
	Mali	16.0 (9.1, 26.5)	14.1 (8.1, 23.4)	18.3 (15.0, 22.1)	15.9 (12.9, 19.4)	14.4 (13.0, 15.9)	14.7 (13.3, 16.3)	-0.6 (-8.1, 6.9)	1.1 (-2.5, 4.8)
	Mauritania	21.4 (9.9, 40.0)	23.5 (11.3, 42.7)	16.8 (13.7, 20.4)	16.8 (13.7, 20.4)	17.2 (15.6, 19.0)	17.2 (15.5, 19.0)	6.3 (-9.4, 22.1)	-0.4 (-4.1, 3.3)
	Nigeria	--^c^	--^c^	--^c^	--^c^	--^c^	--^c^	--^c^	--^c^
	Pakistan	44.6 (33.4, 56.4)	40.2 (30.3, 51.0)	38.1 (31.5, 45.2)	33.8 (27.6, 40.7)	33.7 (30.9, 36.6)	34.5 (31.7, 37.5)	5.7 (-4.9, 16.2)	-0.7 (-7.4, 6.0)
	Rwanda	13.5 (9.6, 18.6)	8.7 (6.4, 11.7)	13.6 (11.4, 16.1)	8.6 (7.2, 10.2)	7.9 (7.0, 8.8)	8.9 (8.0, 9.9)	-0.2 (-3.0, 2.6)	-0.3 (-2.0, 1.4)
	South Africa	49.8 (41.1, 58.5)	47.0 (38.7, 55.4)	38.4 (32.6, 44.6)	36.2 (30.3, 42.5)	38.7 (36.3, 41.2)	39.1 (36.6, 41.6)	7.9 (-0.8, 16.5)	-2.9 (-9.6, 3.7)
	Timor-Leste	58.8 (39.1, 76.1)	54.3 (34.9, 72.5)	59.4 (54.2, 64.3)	52.6 (47.7, 57.5)	57.4 (54.8, 59.9)	58.5 (56.0, 60.9)	-4.2 (-24.0, 15.4)	-5.9 (-11.0, -0.5)^d^
	Uganda	14.1 (8.9, 21.5)	8.3 (5.3, 12.9)	15.5 (12.9, 18.6)	12.1 (9.9, 14.7)	10.2 (9.1, 11.4)	11.0 (9.8, 12.2)	-2.6 (-6.6, 1.3)	1.1 (-1.4, 3.7)

Abbreviation: CI–confidence interval

^a^ Adjusted for: age group, residence, education level, employment status, wealth index, marital status, and health insurance coverage.

^b^ Positive values for adjusted prevalence differences (aPD) indicate higher adjusted prevalence for moderate/severe or mild disability than for no disability; negative aPD indicates higher adjusted prevalence for no disability than for moderate/severe or mild disability.

^c^ Data not available.

^d^ Difference significant at p<0.05.

^e^ Estimate for current use of smokeless tobacco among men with moderate/severe disability in Mauritania was 0.0; therefore, no CIs or aPD were calculated.

^f^ Current use of tobacco products includes combustible/smoked tobacco products, smokeless tobacco products, and any other type of tobacco use included in the Demographic and Health Survey.

### Current use of smokeless tobacco products

In three of the nine (33.3%) countries assessed, the adjusted prevalence of current use of smokeless tobacco products was >10% among women with moderate/severe disability (17.5% [95% CI: 12.0%, 24.8%] in Rwanda, 19.4% [95% CI: 6.0%, 47.6%] in Timor-Leste, and 26.8% [95% CI: 14.4%, 44.3%] in Nigeria; Table 4). A similar pattern was noted for women with mild disability and no disability, with Rwanda, Timor-Leste and Nigeria being the only countries to have a prevalence >10%. For women with a mild disability, the proportion who reported current use of smokeless tobacco products in Rwanda, Timor-Leste, and Nigeria was 17.0% (95% CI: 13.7%, 20.8%), 18.7% (95% CI: 11.0%, 30.1%), and 26.5% (95% CI: 15.7%, 41.3%), respectively; for women with no disability, the prevalence was 16.9% (95% CI: 15.7%, 18.2%), 18.5% (95% CI: 17.1%, 19.9%), and 26.6% (95% CI: 24.1%, 29.2%), respectively. Relative to women with moderate or severe disability, women with no disability in Uganda used smokeless tobacco more (aPD: -0.4 pct pts), with the remaining countries showing no significant difference. This finding was also consistent for Uganda when assessed for women with a mild disability compared with no disability (aPD: -0.5 pct pts). In Haiti, however, women with mild disability reported a higher prevalence of current use of smokeless tobacco products than women with no disability (aPD: 1.5 pct pts).

Among men, in three out of eight (37.5%) countries assessed, the adjusted prevalence of current use of smokeless tobacco products was >10% for those with a moderate/severe disability, mild disability, and no disability (Table 5). The prevalence of men with a moderate/severe disability who currently use smokeless tobacco was 14.7% (95% CI: 8.3%, 24.8%), 19.1% (95% CI: 5.3%, 50.1%), and 27.3% (95% CI: 14.9%, 44.5%) in Pakistan, Rwanda, and Timor-Leste, respectively. These three countries also had prevalence estimates >10% for current smokeless tobacco use among men with mild disability (17.1%, 19.2%, 23.7%, respectively) and no disability (14.2%, 19.2%, 21.5%, respectively); all other countries had prevalence estimates <10%. Like the findings for women, in Uganda, men with no disability had a higher prevalence of current use of smokeless tobacco products than men with a moderate/severe disability (aPD: -1.4 pct pts). No significant differences between men with mild disability and no disability were noted in any of the countries assessed.

### Current use of tobacco products (i.e., smoked and/or smokeless)

The adjusted prevalence for current use of tobacco products among women with a moderate/severe disability, mild disability, and no disability varied across countries, with a median of 1.9% (range = 0.1% [Mali], 11.2% [Pakistan]), 3.1% (range = 0.7% [Nigeria], 13.2% [South Africa]), and 2.3% (range = 0.5% [Nigeria], 8.9% [South Africa]), respectively. Of the countries assessed, Pakistan was the only country with a prevalence of current use of tobacco products >10% among women with moderate/severe disability (11.2%; 95% CI: 7.7%, 16.1%) (Table 4). Similarly, South Africa was the only country with a prevalence >10% for current use of tobacco products among women with a mild disability (13.2%, 95% CI: 9.9%, 17.3%). For women with no disability, no country had a prevalence >10% of current use of tobacco products. Haiti was the only country where women with a moderate/severe disability had a significantly higher prevalence of current use of tobacco products than women with no disability (aPD: 3.9 pct pts). In contrast, in Mali, women with moderate/severe disability had a significantly lower prevalence of current use of tobacco products than women with no disability (aPD: -1.0 pct pt). In three of the nine countries assessed, a significantly higher proportion of women with a mild disability currently used tobacco products than those with no disability (Haiti [aPD: 2.0 pct pts], South Africa [aPD: 4.3 pct pts], and Timor-Leste [aPD: 3.3 pct pts]). Uganda was the only country where a higher proportion of women with no disability reported current use of tobacco products than those with a mild disability (aPD: -0.5 pct pts).

For men with moderate/severe disability, the median adjusted prevalence for current use of tobacco products was 18.9%; Uganda (8.3; 95% CI: 5.3%, 12.9%) and Timor-Leste (54.3; 95% CI: 34.9%, 72.5%) had the lowest and highest prevalence estimates, respectively (Table 5). The median prevalence of current use of tobacco products for men with mild disability and no disability were similar to those with moderate/severe disability, at 16.4% and 16.0%, respectively. Moreover, among men with a mild disability and no disability, Timor-Leste had the highest prevalence of current use of tobacco products at 52.6% (95% CI: 47.7%, 57.5%) and 58.5% (95% CI: 56.0%, 60.9%), respectively; Rwanda had the lowest prevalence of current use of tobacco products at 8.6% (95% CI: 7.2%, 10.2%) and 8.9% (95% CI: 8.0%, 9.9%), respectively. Timor-Leste was the only country where the proportion of men with a mild disability who currently used tobacco products was significantly lower than those with no disability (aPD: -5.9 pct pts). No significant difference in the prevalence of current use of tobacco products between disability status groups was noted for the other countries.

## Discussion

While current tobacco product use among people with disabilities varied for countries included in our study, with few exceptions, the prevalence of current tobacco product use was similar across disability status groups. This finding is similar to studies by Liu et al. (2013) and Choi et al. (2020) which found no significant differences in smoking between older Canadians with and without a mobility disability or South Korean adults with and without physical disabilities, respectively [16, 17]. However, this finding differs from those of studies that used a broader definition of disability that includes multiple disability types; a multi-country study of 21 European countries and several single-country studies from Australia, Indonesia, the UK, and the United States found that, in general, tobacco use was higher for adults with disabilities compared with their counterparts without a disability [5, 6, 12–15, 19, 30, 31]. We noted two previous studies that reported on tobacco use among adults with disabilities in countries included in the current study (Nigeria and Rwanda); however, small sample sizes and lack of comparison groups in these studies preclude us from making meaningful comparisons with them [20, 21]. It is worth mentioning that six of the nine countries included in our study are from sub-Saharan Africa, a region that generally has a lower prevalence of tobacco use relative to countries in other regions of the world [9]. Additional research from geographically and otherwise diverse countries is warranted to determine whether our findings extend beyond the countries included in this study.

We used the WG-SS questions to measure disability, which allowed for examination of disability severity (e.g., mild, moderate/severe). We found some differences in tobacco use by disability severity; for example, in Haiti, a higher proportion of women with mild and moderate/severe disability currently used tobacco products, and in Timor-Leste, a lower proportion of men with a mild disability used tobacco than those without a disability. Like some previous studies, we noted a pattern in some but not all countries (e.g., among women in South Africa and Timor-Leste) wherein people with mild disability had a higher prevalence and people with more severe disability had a comparable or lower prevalence of tobacco use when compared with people without a disability [32–34]. Part of the reason may be that people with more severe disability are more likely to be reliant on caregivers for daily functioning, and therefore may have limited access or opportunity to use or purchase tobacco; whereas those with mild disability may be more similar to those without a disability in terms of access and ability to use tobacco [35]. Further exploration of the reasons underlying this pattern is an area for future work. Consideration of disability, particularly at different levels of severity, can help countries plan how to better direct resources to those in most need and ensure equitable access to tobacco cessation programs and services [36].

Smoking cessation services and treatments available for the general population may not work as well for people with disabilities, and thus, a combination of modalities that have been adapted or tailored, taking into account the unique needs of people with disabilities, are suggested [37–39]. Successful tobacco cessation programs for people with disabilities, such as the Living Independently from Tobacco program, provide social support through group settings and facilitators who help tailor the program to individual needs of participants [39, 40]. Any materials provided must be made available in a range of accessible formats (e.g., in large print for those with visual impairment or in language understandable to those with cognitive impairment) [38]. Additional strategies that may aid people with disabilities in increasing readiness to quit and achieving or maintaining abstinence include: (1) education on the relationship between tobacco use and further disability; (2) promotion of healthy alternatives to activities limited because of a disability; and (3) management of underlying or co-occurring conditions such as anxiety, depression, or pain [39, 41–43]. Disability service organizations or care providers may be prime sites for tobacco cessation programs because their experience in disability-related matters provides them a better understanding of the complexities surrounding disability; however, these organizations and caregivers may need further training on evidence-based tobacco cessation interventions and treatments [38, 44].

Because people with disabilities tend to utilize the healthcare system more frequently than people without disabilities [30, 45], healthcare providers may also be in a unique position to offer help to quit tobacco or provide linkages to services to people with disabilities. People with disabilities who smoke receive quit advice from and discuss treatment options with their healthcare providers at slightly higher rates than people without disabilities [30]. Yet people with disabilities also experience many barriers to accessing health care services, which worsen with increasing severity of disability [46]. These barriers include inaccessible equipment and facilities (i.e., exam rooms that are too small to maneuver a wheelchair), problems with transportation to appointments, and difficulties paying for transportation or health care services [45–48]. People with disabilities may face negative attitudes of staff [45, 46] or have to educate their provider about their disability and leave appointments without having their health-related behaviors addressed or their needs met [47, 48]. Public health initiatives or trainings promoting the awareness of such barriers encountered by people with disabilities and involving them in the design and implementation of tobacco control programs might not only eliminate many of these barriers but also provide an opportunity to broadly reduce health disparities often experienced by this population.

People with disabilities can and should have the same right to health, free from tobacco, as people without disabilities. This vision is supported by two global treaties: the WHO Framework Convention on Tobacco Control, which reaffirms the right of all people to the highest standard of health through protections from tobacco; and the Convention on the Rights of Persons with Disabilities, which calls for the full and equal enjoyment of rights and freedoms by people with disabilities [49, 50]. Global tobacco control, disability justice, and health equity efforts may be enhanced through a multi-sectoral approach that engages a network of relevant stakeholders that includes people with disabilities, disability rights organizations, and tobacco control advocates [51].

This study is subject to several limitations. First, data used in this study are cross-sectional, so we are unable to determine causality (i.e., whether having a disability led to tobacco use or vice versa). Several studies have shown an increased risk of disability among people who smoke [52–54]; however, it is important for people who have a disability and use tobacco to quit regardless of which came first. Second, information on disability status was reported by a selected household member, who may or may not be well informed about the disability status of all others in their household. Previous research on the DHS disability module found only small differences between self- and proxy-report, mostly related to the type of disability (e.g., whether the disability was apparent or non-apparent) [55]. Third, information on tobacco use was self-reported, which could lead to recall or social desirability biases. Fourth, multiple comparisons were performed, so it is possible some significant results observed may be due to chance. Fifth, while tobacco use has been linked with mental health conditions such as anxiety and depression [13], it was beyond the scope of our study to investigate the relationship between disability, mental health, and tobacco use. Lastly, while some recent studies have demonstrated disparities in e-cigarette use between adults with disabilities and those without [56–58], little or no DHS data were available on e-cigarettes or other non-combustible nicotine and tobacco products (e.g., heated tobacco products, nicotine pouches, etc.); this is an area for future work. Despite these limitations, this study adds to the global body of literature by using nationally representative survey data which allows for comparability over time as well as standard measures of disability and tobacco use that are more representative across a global spectrum than what has been studied previously.

## Conclusions

People with disabilities make up a considerable portion of the global population; in our study, the median prevalence of combined mild and moderate/severe disability was approximately 15%, or almost one out of every six adults. In most countries assessed, similar proportions of men and women with disabilities used tobacco as their counterparts without disabilities; however, this finding may not be generalizable globally since most countries included in our study are in sub-Saharan Africa, where tobacco use prevalence is relatively low. Nevertheless, it is important to consider the needs of people with disabilities in tobacco prevention, control, and cessation efforts; otherwise, a substantial share of the population may not be able to benefit from such programs, interventions, or policies.
